# Rationality and the illusion of choice

**DOI:** 10.3389/fpsyg.2014.00104

**Published:** 2014-02-12

**Authors:** Jonathan St. B. T. Evans

**Affiliations:** School of Psychology, University of PlymouthPlymouth, UK

**Keywords:** rationality, decision making, folk psychology, illusion of control, reasoning

## Abstract

The psychology of reasoning and decision making (RDM) shares the methodology of cognitive psychology in that researchers assume that participants are doing their best to solve the problems according to the instruction. Unlike other cognitive researchers, however, they often view erroneous answers evidence of irrationality rather than limited efficiency in the cognitive systems studied. Philosophers and psychologists also talk of people being irrational in a special sense that does not apply to other animals, who are seen as having no choice in their own behavior. I argue here that (a) RDM is no different from other fields of cognitive psychology and should be subject to the same kind of scientific inferences, and (b) the special human sense of irrationality derives from folk psychology and the illusory belief that there are conscious people in charge of their minds and decisions.

## INTRODUCTION

Two fields stand out as different within cognitive psychology. These are the study of reasoning, especially deductive reasoning and statistical inference, and the more broadly defined field of decision making. For simplicity I label these topics as the study of reasoning and decision making (RDM). What make RDM different from all other fields of cognitive psychology is that psychologists constantly argued with each other and with philosophers about whether the behavior of their participants is *rational* (see Cohen, 1981; Stanovich and West, 2000; Elqayam and Evans, 2011). The question I address here is why? What is so different about RDM that it attracts the interests of philosophers and compulsively engages experimental psychologists in judgments of how good or bad is the RDM they observe.

Let us first consider the nature of cognitive psychology in general. It is branch of cognitive science, concerned with the empirical and theoretical study of cognitive processes in humans. It covers a wide collection of processes connected with perception, attention, memory, language, and thinking. However, only in the RDM subset of the psychology of thinking is rationality an issue. For sure, *accuracy* measures are used throughout cognitive psychology. We can measure whether participants detect faint signals, make accurate judgments of distances, recall words read to them correctly and so on. The study of non-veridical functions is also a part of wider cognitive psychology, for example the study of visual illusions, memory lapses, and cognitive failures in normal people as well as various pathological conditions linked to brain damage, such as aphasia. But in none of these cases are inaccurate responses regarded as *irrational*. Visual illusions are attributed to normally adaptive cognitive mechanisms that can be tricked under special circumstances; memory errors reflect limited capacity systems and pathological cognition to brain damage or clinical disorders. In no case is the *person* held responsible and denounced as irrational^1^.

Even in the psychology of thinking, the same approach prevails in many topic areas. For example, when we give people longer letter strings they increasing fail to find anagrams. We do not say that failing to solve a long anagram problem is irrational; indeed it would seem quite anomalous to do so. In fact, in the broader field of problem solving generally, despite obvious similarities with RDM, there is much measurement of error but no debate about rationality. We measure performance errors to investigate psychology mechanisms and their design limitations but not to declare people irrational as result. But if the psychology of problem solving needs no rationality debate, why is it that the study of RDM does?

## NORM-REFERENCING IN COGNITIVE PSYCHOLOGY

A clear correlate of rationality debating within cognitive psychology is the prevalence of *norm-referencing*. In most of cognitive psychology there is little or no debate about what constitutes an error. A signal is present or not and hence detected or not by the participants’ judgment; a word recalled was either present or absent in the list of words presented to the participant; an anagram offered either uses the letters presented or it does not. But the study of RDM is different in this respect. In these fields, experimenters need to apply a *normative theory* in order to decide whether an error has been made. If we divide cognitive psychology into fields that are norm-referenced and those that are not, there is an almost perfect correlation with the presence of rationality judgments.

It is important to note that normative theories are not psychological theories and that they derive from disciplines outside of psychology. For example, the dominant theory of rational decision making was derived from the disciplines of economics and mathematics (von Neumann and Morgenstern, 1944) and first introduced to psychologists by Edwards (1954). Study of decisions made under uncertainty, and the assessment of risk became a mainstream topic for psychologists who attempted to assess conformity to rational principles, as defined by economists and mathematicians. A spin-off from this was to study people’s intuitive grasp of statistical principles derived from the probability calculus, such as Bayes’ theorem. While early assessment of people’s intuitive statistical abilities were optimistic (Peterson and Beach, 1967), this soon changed when Tversky and Kahneman (1974) launched their heuristics and biases program in early 1970s (for later reports, see Kahneman et al., 1982; Gilovich et al., 2002).

Wason (1960, 1966, 1968) and Wason and Johnson-Laird (1972) famously attributed irrationality to his participants based on their frequent failure to solve his 2-4-6 and selection task problems (see Evans, 2002, for quoted examples). He described a verification bias, more generally known as confirmation bias, which he suggested was irrational as it failed to comply with Popper’s strictures for good scientific thinking. None of this has stood the test of time as his verification bias account has been discredited for both tasks (see Evans, 2007a) and Popper’s philosophy of science has been strongly challenged by Bayesian critics (Poletiek, 2001; Howson and Urbach, 2006). In a sense, however, that is beside the point. People were considered irrational because they appeared to violate a popular normative theory of the time (Popper, 1959). Similarly, studies of deductive reasoning from the 1980s onward have shown people to be illogical (Evans, 2007a; Manktelow, 2012) but again the use of standard logic has been challenged (e.g., Oaksford and Chater, 2007).

It is evident that the need to apply a normative theory creates problems that are not present in other parts of cognitive psychology because we can debate whether such theories are correctly formulated or appropriately applied. However, it is far from obvious to me why in itself this should lead to a rationality debate. Why is a person wrongly identifying a face merely mistaken, while a person failing to maximize utility or making a logical error irrational? As we have seen, in most parts of cognitive psychology, evidence of error is not seen as evidence of irrationality. In fact, it seems quite ludicrous to suggest, for example, that someone falling prey to a standard visual illusion is being irrational. So there must be more to this problem than simply the ambiguity involved with norm referencing.

## RATIONALITY AND VOLITION

A pigeon that learns to peck at a key in order to obtain food pellets can be described as *instrumentally rational, *that is, acting in such a way as to achieve its goals. Instrumental rationality is also known sometimes as personal or individual rationality (Stanovich, 1999). In fact, the argument can be made that animals are *more* instrumentally rational than humans as defined by performance on judgment and decision making tasks (Stanovich, 2013). Humans, with their complex layers of multiple goals and value systems will not always choose correctly according to the immediate goals that the psychologists uses to determine rationality. Of course, we could argue that this is due more to incorrect applications of norm-referencing than superior rationality of animals.

If we consider animals a little more, it becomes clear that there is a curious lack of complementarity between the terms rational and irrational. Animals frequently follow instinctive behavior patterns which conflict with their individual interests, exposing themselves to injury or death in pursuit of the interests of their selfish genes. More accurately, they follow instructions which helped genes to replicate in their environment of evolutionary adaptation at some time in the past. So are animals behaving *irrationally* when they act (by genetic compulsion) in ways that violate their interests as individuals? Surely not, as they have no choice in the matter. As Stanovich (2011), p.3) puts it: “an animal can be arational, but only humans can be irrational.” But if they are not irrational when they act against their interests, in what sense are they rational when they act for them? There is some sense of rationality, applicable to humans, which seems not to apply to non-human animals.

It seems to me that in this important and distinctly human sense of the term, rationality is not simply to do with instrumentality; it is to do with *choice*. I have written elsewhere on the theory that humans have an old mind, animal like in many ways, combined with a new and distinctively human mind (Evans, 2010, in press; see also R’eber, 1993; Epstein, 1994; Evans and Over, 1996; Stanovich, 2004 for examples of many related earlier works along these lines). The rationality of the old mind is very much like the rationality of animals. We, like them, learn habits and procedures from experience that enable us to repeat behaviors rewarded in the past. This provides us and them with a form of instrumental rationality. But new mind rationality is not the slave of the past; as humans we can imagine the futures, conduct thought experiments and mental simulations and choose to act in one way rather than another. We can also (sometimes) manage to override our old minds, inhibiting our wishes to smoke cigarettes, join gambling games and other activities which may feel quite compulsive but conflict the goals that are new mind is setting for our futures. In fact, we are most likely to praise someone as rational when the new mind overrides in this way and conversely quick to condemn as irrational, the people who give way to their basic urges. However, while new mind cognition is volitional that does not mean that the individual is free to choose actions in all circumstances. Our behavior is the product of both old and new minds and so powerful emotions and strong habits may override the choices of the new mind. It is also a mistake to equate the new mind with the conscious person (see Evans, 2010, Chap. 7).

Another issue here lies with the general methodology of cognitive psychology. All cognitive experiments study *intendedly rational behavior*. It is nothing distinctive to RDM that participants are assumed to understand the instructions and be attempting to comply with them. If they were not bothering, then we could not, for example, infer that failure to recall a word reflected a limitation in memory capacity. What is distinctive to RDM is that when people fail to find the correct answer (according to some normative theory) they are often deemed to be irrational. But the method *presupposes* new mind rationality (compliance with instructions, making best effort). How can we both presuppose rationality and then infer irrationality from errors? Researchers in no other fields of cognitive psychology do this, inferring instead cognitive limitations from errors.

There is nothing inherently different about RDM tasks that justifies this difference. If the assumption of intendedly rational behavior is sound for the study of lexical decisions, semantic memory and size constancy, then it is also sound for the study of deductive reasoning, probability judgment and decision making. If RDM researchers can say that people did not really understand the instructions or were not doing their best of comply with them, then why should we assume that they were compliant in studies of the serial position curve? If – as seems much more likely – RDM researchers endorse the cognitive method and share its assumptions, then on what basis can they equate errors with irrationality? Is it the underlying cognitive mechanisms that cause irrational choices, despite the best efforts of the conscious person? But in what sense can a mechanism be said to be irrational? It can be well or badly designed, fit for purpose or not but surely it cannot have rationality.

Stanovich (2011, p. 5) is admirably clear on this point: “… rationality is a personal entity and not a subpersonal one … A memory system in the human brain is not rational or irrational, it is merely efficient or inefficient,”. So it would seem that rationality, in this special human sense, is a property of the *person*. But who or what exactly is the person? It is clearly not be equated with organism as a whole, nor with the brain. So my brain cannot be irrational, and nor can the mind defined as the whole working of the brain in terms of its cognitive processes. In my detailed account of the two minds theory, I describe the person as a construction of the new mind and in many ways an illusory one. The conscious person whom we feel ourselves to be is subject to illusion of control and intentions that have been cleverly demonstrated by researchers in social psychology (see Evans, 2010, Chap. 7).

## FOLK PSYCHOLOGY AND TWO MINDS CONFLICT

I think it is time for me to propose an answer to the puzzle. What is it about RDM that provokes a rationality debate absent in the rest of cognitive psychology? I believe the answer lies in folk psychology, in the ingrained beliefs that we all hold about the human mind and its operation^2^. Folk psychology embodies what I call the Chief Executive Model of the mind (Evans, 2010). We think of ourselves and others as conscious people in charge of our decisions^3^. To be sure there are many automated and unconscious mechanisms responsible for such matters as language processing, pattern recognition, memory retrieval etc. But these are merely slave systems doing our bidding. We, the conscious persons, are still in charge, still calling the shots. This is a powerful illusion, but an illusion nonetheless. There is now much accumulated evidence that we lack knowledge of our mental processes and the reasons underlying our decisions, frequently rationalizing or theorizing about our own behavior (Wilson et al., 1993; Wilson, 2002). The feeling that we are in control and that conscious thought determines actions is also an illusion (Bargh and Ferguson, 2000; Velmans, 2000; Wegner, 2002).

In two minds theory (Stanovich, 2004; Evans, 2010) conflict can easily arise between the goals that are pursued in the new and old minds. Moreover, the cognitive mechanisms for pursuit of goals differ radically, with experiential learning dominating the old mind, and hypothetical thinking the new mind. Two minds conflict is the essential cause of the cognitive biases that are observed in the study of reasoning and decision making. Biases arise from automated and unconscious mechanisms which divert us from solution of the tasks set. Frequently, there is a default intuitive response that leads people into error unless overridden by conscious reasoning (Kahneman and Frederick, 2002; Frederick, 2005; Evans, 2007b; Stanovich, 2011; Thompson et al., 2011). The ability to override such defaults is influenced by a number of factors including confidence in the original answer, cognitive ability and thinking dispositions. But in general, when someone fails to reason correctly according to the instruction it is due to an unconscious or intuitive influence of some kind. They are not choosing to get the answer wrong^4^.

Outside of the laboratory, the behavior that strikes us as irrational is that in which a person experiences a two minds conflict in which the old mind is winning. For example, the heavily obese, compulsive gamblers and alcoholics are treated with very little sympathy in modern society. They are held to be responsible for their own health or financial problems because they could apparently *choose* to be different. Those of us who are not problem gamblers, for example, think it quite *irrational* that people should continue to bet money on casino games like roulette. The normative theory agrees, because all betting systems are based on the fallacious belief that later bets can compensate for earlier ones, whereas each individual bet has an expected loss (Wagenaar, 1988). But from a psychological point of view this normative analysis is not only simplistic but essentially useless in understanding the causes of problem gambling and how to deal with them. Most effective in such cases is cognitive-behavioral therapy which is essentially a two minds treatment (see Evans, 2010, Chap. 8).

## CONCLUSIONS

There is nothing wrong with normative theories in themselves, nor with the tendency to debate which one is appropriate for a particular task. It is useful to have a measurement of error in RDM for the same reason as in other fields of cognitive psychology. If our decisions are suboptimal, for example, we can ask what limitations of our cognitive mechanisms are responsible. Is it a capacity limitation, or lack of experience or relevant learning? I have no problem, for example, agreeing that neglecting base rates in Bayesian inference is an error (Barbey and Sloman, 2007). I do have great difficulty in seeing it as evidence for irrationality, however. If people have not studied statistics, do not know the equation of Bayes’ theorem and are not able to do complicated calculations in their heads, it is not surprising they make errors. But why is this irrational? As Elqayam and Evans (2011) point out, it as though *learning* has been excluded from the equation. We must apparently be able to reason well without relevant training and learning in order to be judged as rational.

The problem lies not in the use of normative systems as such but in equation of conforming to them as an indicator of rational thought. Perhaps this practice is inherited from disciplines like philosophy and economics from which our normative theories derive. But to me it does not justify the treatment of RDM as different from any other field of cognitive psychology. We are still studying intendedly rational behavior and if people make errors it is not because they could have chosen to do otherwise. The belief that people can be irrational in a special sense that does not apply to other animals derives, I believe, from an illusion in folk psychology that there are somehow conscious persons, distinct from their minds and brains, who are in control of their behavior. People are certainly in possession of minds that are limited, inefficient and not always well adapted to the task at hand. So they are not invariably rational in the way that Panglossian authors (e.g., Cohen, 1981) claim, meaning that people are invariably well adapted and optimized. But nor can people be *irrational *either, in the sense derived from folk psychology.

## Conflict of Interest Statement

The author declares that the research was conducted in the absence of any commercial or financial relationships that could be construed as a potential conflict of interest.

## Footnotes

1 I am not saying that judgmental terms are entirely absent in other fields of psychology, for example with regard to false memories and unfounded beliefs. However, I believe that reasoning and decision making are the only topics in which rationality is a central concern.

2 Note that I am not restricting the use of the term “folk psychology” to belief-desire psychology as is common in the philosophical literature.

3 Folk psychology is close to the (largely discredited) interactive dualism of Descartes on this point. If I am right about this, then he was essentially formalizing intuitions about conscious minds that we all share.

4 Stanovich’s (2011) analysis implies that the choice lies within the “rational” thinking dispositions of what he calls the reflective mind. My view is that such dispositions are personality characteristics that are not chosen by the “person”. The fact that, as he correctly claims, such dispositions can be modified by education and training is neither here nor there.
